# Direct Liquid Extraction and Ionization Techniques for Understanding Multimolecular Environments in Biological Systems (Secondary Publication)

**DOI:** 10.5702/massspectrometry.A0095

**Published:** 2021-06-30

**Authors:** Yoichi Otsuka

**Affiliations:** 1 Graduate School of Science, Osaka University, 1–1 Machikaneyama-cho, Toyonaka, Osaka 560–0043, Japan; 2 JST, PRESTO, 4–1–8 Honcho, Kawaguchi, Saitama 332–0012, Japan

**Keywords:** electrospray ionization, direct extraction, mass spectrometry imaging, charged droplet, derivatization

## Abstract

A combination of direct liquid extraction using a small volume of solvent and electrospray ionization allows the rapid measurement of complex chemical components in biological samples and visualization of their distribution in tissue sections. This review describes the development of such techniques and their application to biological research since the first reports in the early 2000s. An overview of electrospray ionization, ion suppression in samples, and the acceleration of specific chemical reactions in charged droplets is also presented. Potential future applications for visualizing multimolecular environments in biological systems are discussed.

## 1. INTRODUCTION

The cell is smallest structural and functional unit of an organism. Cells contain a wide variety of molecules in order to maintain homeostasis. A cell is on the order of tens of micrometers in diameter and several picoliters in volume, and its molecular concentration ranges from 10^−3^ mol to 10^3^ mol per cell.^1)^ Various molecules with different molecular weights and chemical properties interact with each other inside cells to conduct metabolic reactions, and their composition and quantity change dynamically according to the state of the cell.

Liquid–liquid phase separation is based on intracellular molecular interactions and has recently attracted much attention for its potential to elucidate spontaneous biological processes in non-equilibrium conditions.^2)^ Phase separation results in the formation of membrane-free compartments containing aggregated biomolecules in the cell. This spontaneous aggregation is reported to cause neurodegenerative diseases, cancers, and infections by generating a microenvironment in which certain components are enriched and accelerate or inhibit biochemical reactions.^3,4)^ Therefore, understanding the heterogeneity of the chemical composition of biological tissues, which are composed of cellular networks, is important for elucidating the pathogenesis of diseases and for developing advanced diagnostic technologies in the future.

Mass spectrometry (MS) is a powerful analytical technique that captures detailed information about ions. If a molecule in a solid or liquid can be converted into a gas-phase ion by an ionization technique, its *m*/*z* and intensity can be measured, and the chemical structure of the molecule can be identified and quantitatively evaluated.

There are various ionization techniques, of which electrospray ionization (ESI)^5)^ is one of the most popular soft ionization techniques for biomolecules. Application of a high electric field to a sample solution generates microdroplets of the charged sample solution. During evaporation of these charged droplets, the molecules in the droplets are freed from solvation and form gas-phase ions. Given that biomolecules such as metabolites, lipids, and proteins can be ionized without fragmentation, the combination of ESI and MS is an extremely important analytical technique for life science research. ESI can ionize not only single molecules but also protein complexes, and thus ESI has been used to study molecular interactions.^6–9)^

**Figure 1:**
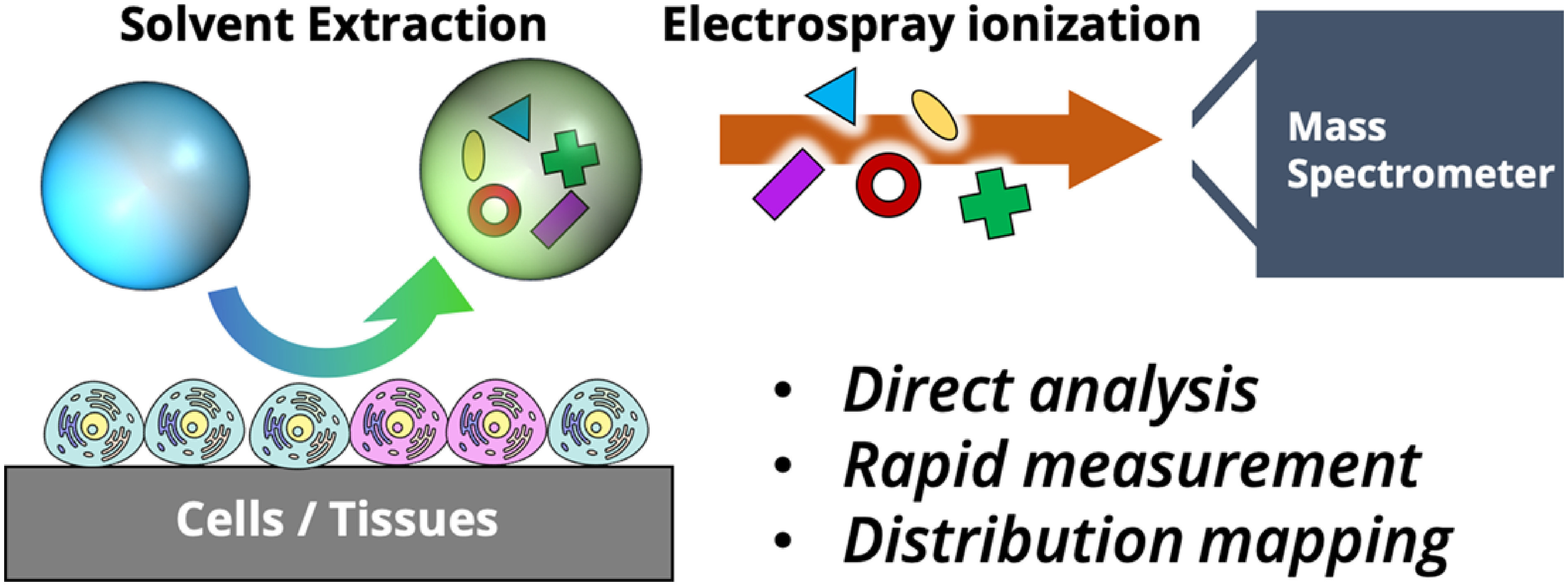
Fig. 1. Schematic illustration of direct extraction using solvents, and direct liquid extraction ionization (DLEI) using electrospray ions. Biological components are extracted by applying a solvent to a micro region of tissue or cells on a substrate. By subjecting the extracted solution to electrospray ionization, multiple components are soft-ionized and introduced into a mass spectrometer. This method does not require any pretreatment and allows rapid mass analysis of chemical information regarding the tissues and cells. In addition, by scanning the sampling area on the sample, the distribution of chemical components can be imaged.

The development of direct liquid extraction and ionization (DLEI), which is a combination of direct extraction and ESI using small amount of solvent, and the application of DLEI to biological samples have gained momentum since the early 2000s. Methods have been developed that use a minute volume of solvent to locally extract and desorb sample components under atmospheric conditions, then immediately ionize and guide them to a mass spectrometer. Because these methods basically require no sample pretreatment or introduction into a vacuum chamber, they are characterized by their ability to conduct MS of multiple components in a sample rapidly. In addition, these methods are highly compatible with mass spectrometry imaging (MSI). Scanning the ion source on the sample in two dimensions provides mass spectra associated with positional information on the sample. The dataset has a multidimensional structure, and the distribution of the ionized components can be visualized by connecting the signal intensity at a specific *m*/*z* with the coordinate information of the sample off-line (Fig. 1).

Typical examples of ionization methods other than ESI are matrix-assisted laser desorption ionization (MALDI) and secondary ionization mass spectrometry (SIMS). Both techniques can also be used for MSI.

In MALDI, sample components with a matrix can be desorbed and ionized by the irradiation of laser light. Spatial resolution can be reduced to a few micrometers compared with DLEI, making high-definition MSI possible. However, matrix application might affect the measurement results, and matrix ions appear in the mass spectrum sometimes make data analysis difficult.

SIMS does not usually require the application of a matrix, and the sample components can be desorbed and ionized by bombarding the sample with an accelerated ion beam. By focusing the ion beam, MSI with a higher spatial resolution (about 0.1 μm) compared with MALDI or DLEI is possible. By controlling the ion beam fluence, it is possible to analyze the top surface of the sample as well as its depth profile. However, fragment ions are produced when measuring biomolecules.

In this paper, an overview of ESI is described first, which was developed over 30 years ago and is constantly improving. Next, the introduction of DLEI and its applications to biomedical measurements is noted and the extent to which intrinsic multidimensional chemical distribution information in cells and tissues has been investigated is described. In addition, the ion suppression and derivatization, which are necessary for the advancement of DLEI, is described. Finally, the unique chemical reactions occurring in charged droplets are introduced, and possibilities for the future advancement of DLEI are discussed.

In addition to the techniques described in this paper, there is a wide range of atmospheric pressure sampling ionization techniques and applications. For example, several methods for sample desorption by laser irradiation and post-ionization by ESI have been proposed, depending on the wavelength, type of sample pretreatment,^10–14)^ and the application to biomolecules^15–17)^ and MSI.^14,18–20)^ The spatial resolution of sampling depends on the size of the laser spot, and ionization can be performed during chemical reactions by adding reactive reagents to the desorbed ESI solution. In addition, various techniques can be used for bioinformatics measurements, such as remote sampling ionization^21–26)^ and rapid sampling ionization techniques.^27–30)^ Because this study focuses on DLEI and related research, we refer the reader to comprehensive reviews of atmospheric pressure sampling ionization,^31–33)^ chemical measurements,^34)^ metabolite measurements^35)^ and medical measurements.^36,37)^

## 2. OVERVIEW OF ELECTROSPRAY IONIZATION

### 2.1 Generation of charged droplets

**Figure 2:**
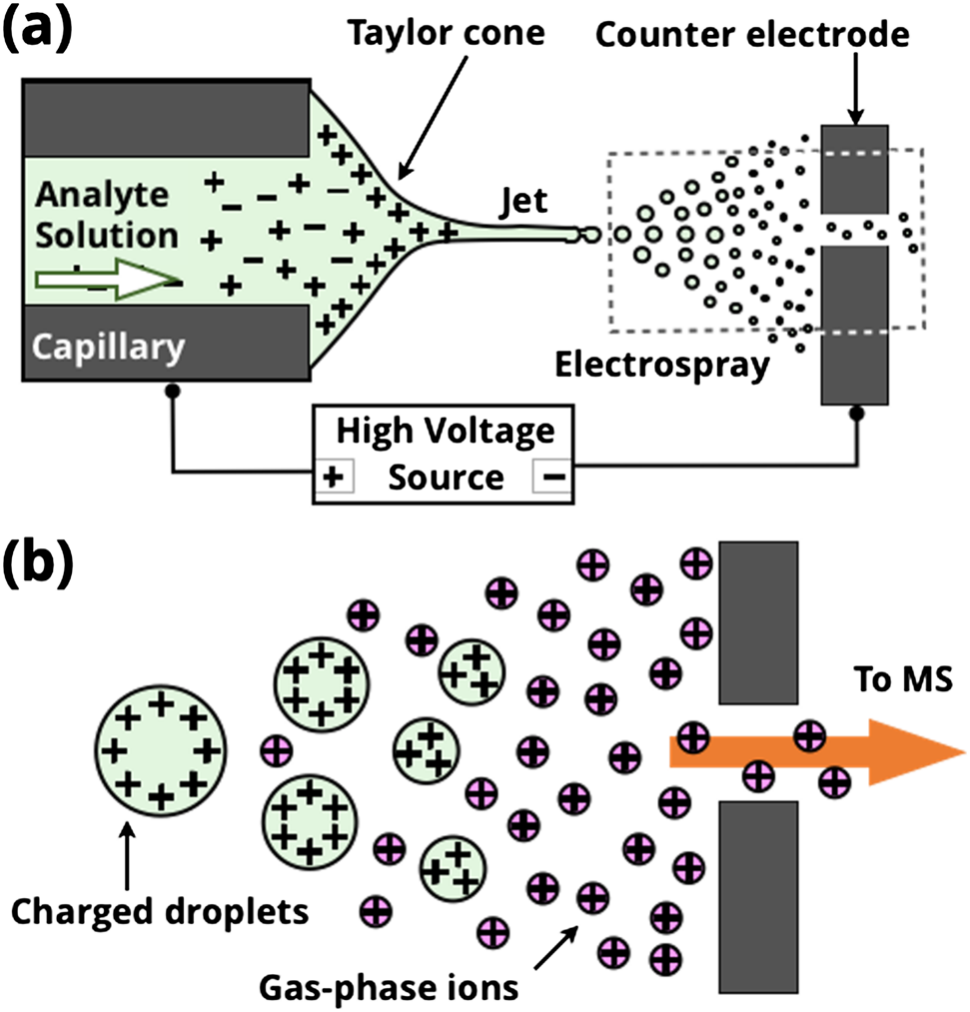
Fig. 2. Schematic diagram of electrospray ionization. A Taylor cone and charged droplets are generated by applying a high voltage to a sample solution as it flows through a microchannel. The charged droplets dry as they fly toward a counter electrode, and the components inside the droplets are soft-ionized.

Fenn *et al.* were the first to apply electrospray (ES)^38)^ to MS and recognized that ESI represented a technological breakthrough for obtaining information on a wide variety of molecular ions.^5)^ A schematic diagram of ESI is shown in Fig. 2. A sample solution flowing through a capillary with micro-scale openings is charged by applying a high voltage through the metal capillary or a metal wire inserted into the glass/fused silica capillary. The high electric field that forms between the capillary and the counter electrode, along with the polarization of the solution, cause the solution to adopt a cone shape, which is called a Taylor cone. When the electric field is sufficiently high, the Coulomb repulsive force associated with the charge at the apex of the Taylor cone exceeds the surface tension, and a fine columnar jet is formed from the tip of the cone in the direction of the electric field. The jet eventually breaks and micro-scale charged droplets are generated (Fig. 2(a)). The electrical potential required to generate an ES is estimated using Eq. (1): 
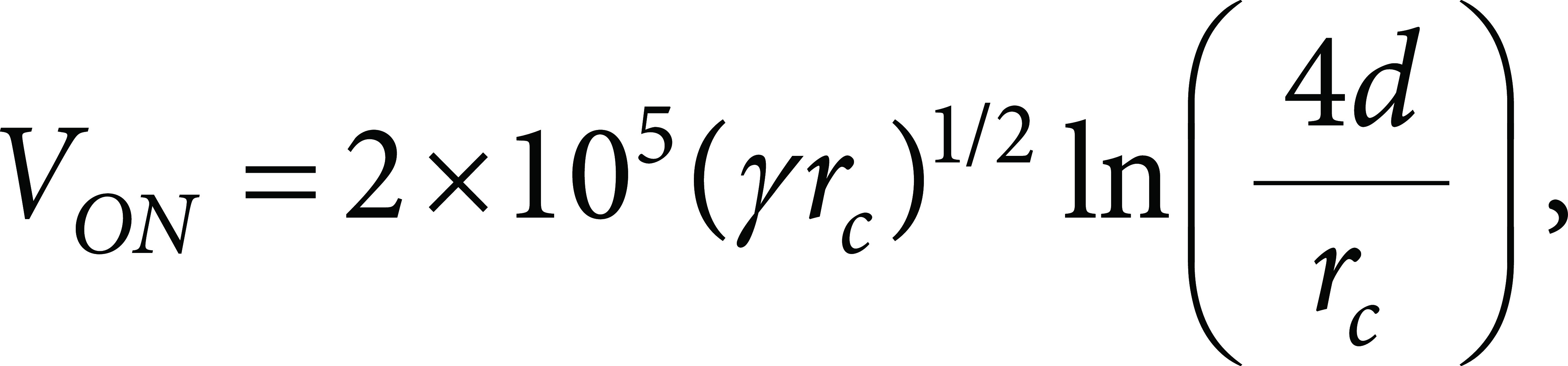
(1) where *V_ON_* is the voltage applied to the emitter, γ is the surface tension of the solvent, *r_c_* is the radius of the capillary, and *d* is the distance between the emitter and the counter electrode. Therefore, *V_ON_* is the appropriate voltage required to produce ES, which varies depending on the physicochemical properties of the sample solution and the spatial arrangement of the emitter.^39)^

**Figure 3:**
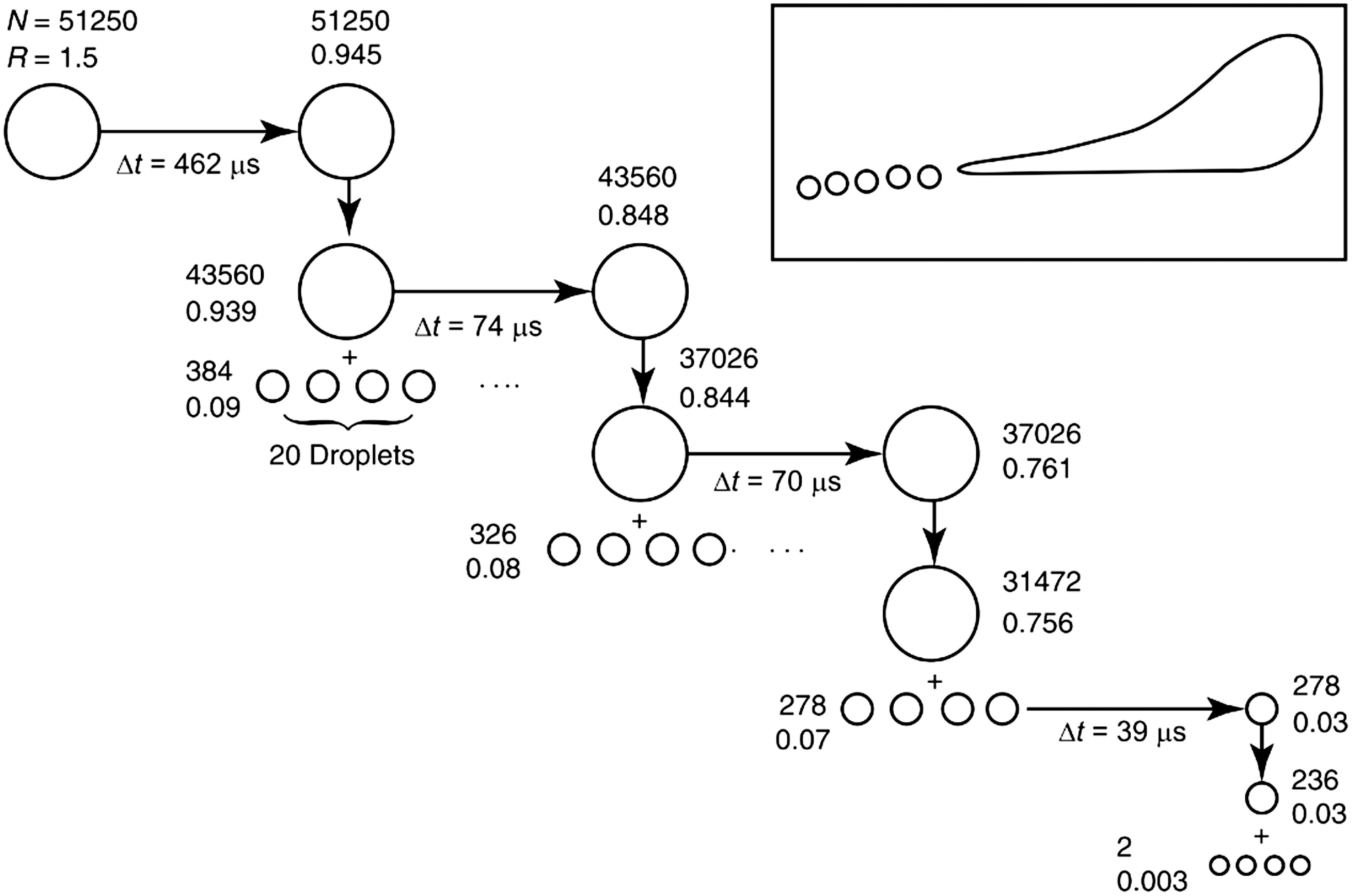
Fig. 3. Schematic diagram of the time evolution of charged droplets generated by electrospray ionization, where *N* is the number of charges, Δ*t* is the elapsed time, and *R* is the diameter (μm). During the drying process, the charged droplet shrinks in the order of μsec, resulting in the release of nanoscale charged droplets. The inset is a representation of droplet formation based on the photograph of experiment. Reproduced with permission from Ref. 39. Copyright (1993) American Chemical Society.

The charged droplet flies toward the ion inlet of the mass spectrometer according to the electric field between the capillary and the counter electrode, and the flow of inert gas or air supplied with various configurations depending on the MS vendor to facilitate drying of the droplets (Fig. 2(b)). During this process, the droplets shrink as the solvent evaporates, and the surface charge density of the droplets increases. When the Coulomb repulsive force between the surface charges overcomes the surface tension of the droplets, Coulomb splitting (also called Coulomb explosion) occurs, and smaller charged droplets are generated one after another from the original droplets. The initial charged droplets have a diameter on the μm scale, from which multiple charged droplets with diameters ranging from 3 nm to 90 nm are successively generated within about 645 μs^40,41)^ (Fig. 3). Coulomb splitting occurs when the charge of a droplet reaches the Rayleigh equation^42)^ condition^43)^ (Eq. (2)): 
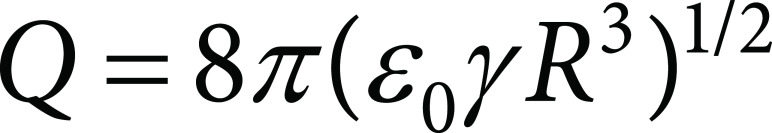
(2) where *Q* is the charge of the droplet, γ is the surface tension of the solvent, *R* is the diameter of the droplet, and ε_0_ is the electrical permittivity.

### 2.2 Dynamic changes in charged droplets

The size of the charged droplets generated by ESI changes dynamically on a microsecond time scale during the drying process. The first measurements of charged droplets using fluorescence and phase Doppler anemometry (PDA), in 1993, used droplets of Rhodamine B in acetonitrile solution. These droplets were about 7 μm in diameter immediately after ejection from the capillary, then decreased to less than 1 μm as the distance from the capillary increased (drying time increased). The droplets flew at a speed of about 5 m/s. For an initial droplet concentration of 10 μM, it was estimated that the solute would be concentrated to 10 mM after several ms of drying.^44)^

A study of the effects of composition, pH, and polarity of the applied voltage on the droplet size of an acetonitrile/water mixture reported that the size of the charged droplet and the subsequent drying process were independent of the polarity of the applied voltage, and that the droplet size decreased with the amount of volatile solvent in the droplet.^45)^

Electrospray studies of an ethanol solution showed that the morphology (mode) of the electrospray changed when the applied voltage was increased from 4.2 to 6.3 kV. The average droplet size at a distance of 10 mm from the capillary decreased from about 23 to about 8 μm, and the droplet velocity in the direction of the counter electrode increased from about 4.8 to 33 m/s.^46)^ Furthermore, the physical properties of charged droplets change, and a decrease in temperature,^47)^ a decrease in the pH of acidic solvents,^48)^ and fractionation of solvent composition^49)^ during the reduction process of charged droplets using laser-induced fluorescence spectroscopy^50)^ have been reported.

### 2.3 Models for ion generation from charged droplets

**Figure 4:**
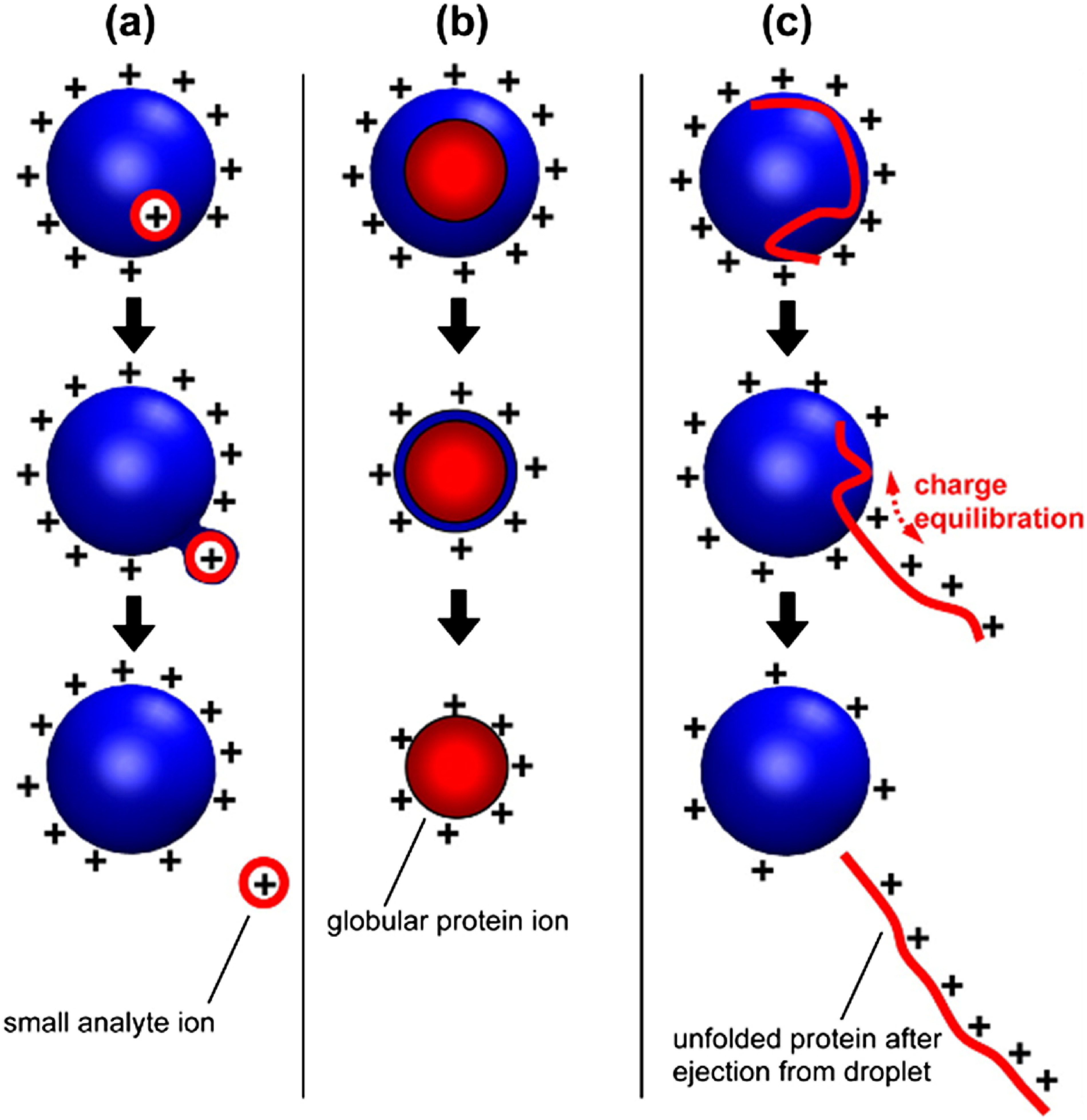
Fig. 4. Schematic diagram of ion generation in ESI. (a) Ion evaporation model (IEM), (b) ion residual model (CRM), and (c) chain ejection model (CEM). Reproduced with permission from Ref. 52. Copyright (2013) American Chemical Society.

The ion evaporation model (IEM) and the charge residue model (CRM) are representative models of ion generation by ESI. During the evaporation process of a charged droplet in IEM (Fig. 4(a)), molecules in the droplet are ejected from the droplet surface as molecular ions and are transformed into gas-phase ions. This occurs when the Coulomb repulsion due to the increase in the charge density on the droplet surface compensates for the energy required to expand the droplet surface for the ions to be ejected. The rate constant for droplet ejection is given in Eq. (3)^51,52)^: 
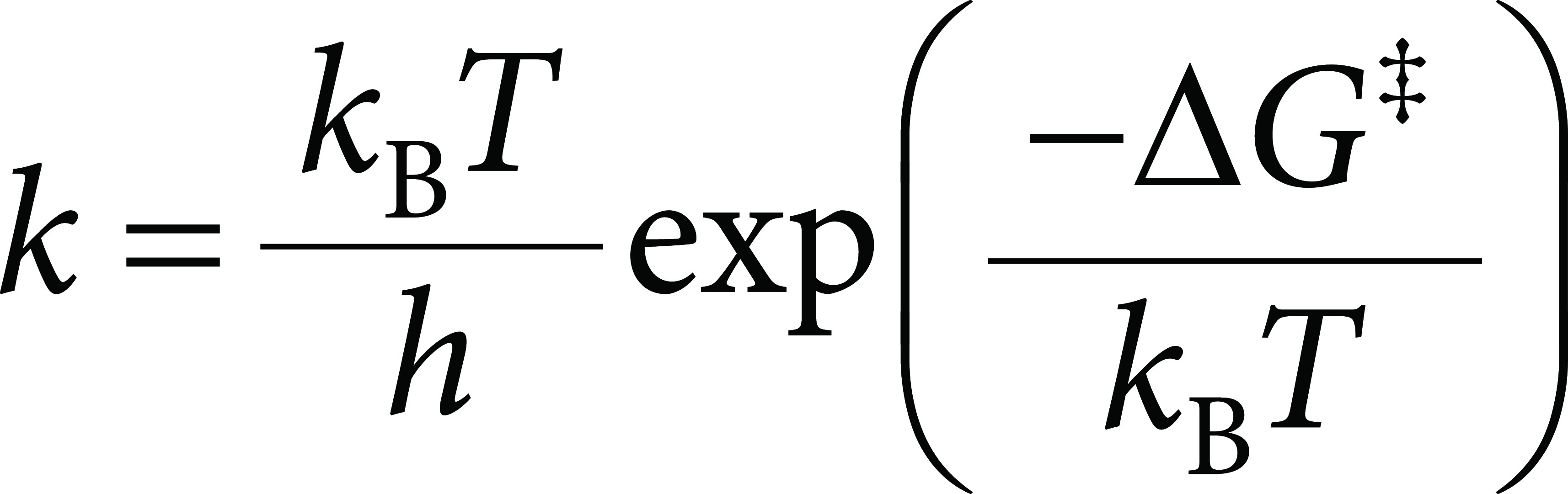
(3) where Δ*G*^‡^ is the Gibbs energy, *k*_B_ is Boltzmann’s constant, *h* is Planck’s constant, and *T* is temperature. Previous reports have shown that the ionization efficiency of IEM varies with the concentration of the analyte in solution and with surface activity.^53)^

In the CRM (Fig. 4(b)), molecules remain in the charged droplet while the solvent evaporates, and finally the charge in the droplet is transferred to the molecules, generating molecular ions.^54)^ The ionization of proteins often produces multiply-charged ions. Multiple protons adsorbed to a protein are likely due to CRM, given that the number of charges in a multivalent globular protein is the same as the number of charges in a charged droplet of the same diameter at the Rayleigh limit.^55)^

In addition to the above two models, the chain ejection model (CEM) was proposed as an ionization mechanism for denatured proteins (Fig. 4(c)). Molecular dynamics simulation shows that molecular chains are released from the surface of charged droplets in a stepwise manner as the protein is denatured, and multivalent ions are formed in the process.^56)^

## 3. DEVELOPMENT AND APPLICATION OF DIRECT LIQUID EXTRACTION AND IONIZATION

**Figure 5:**
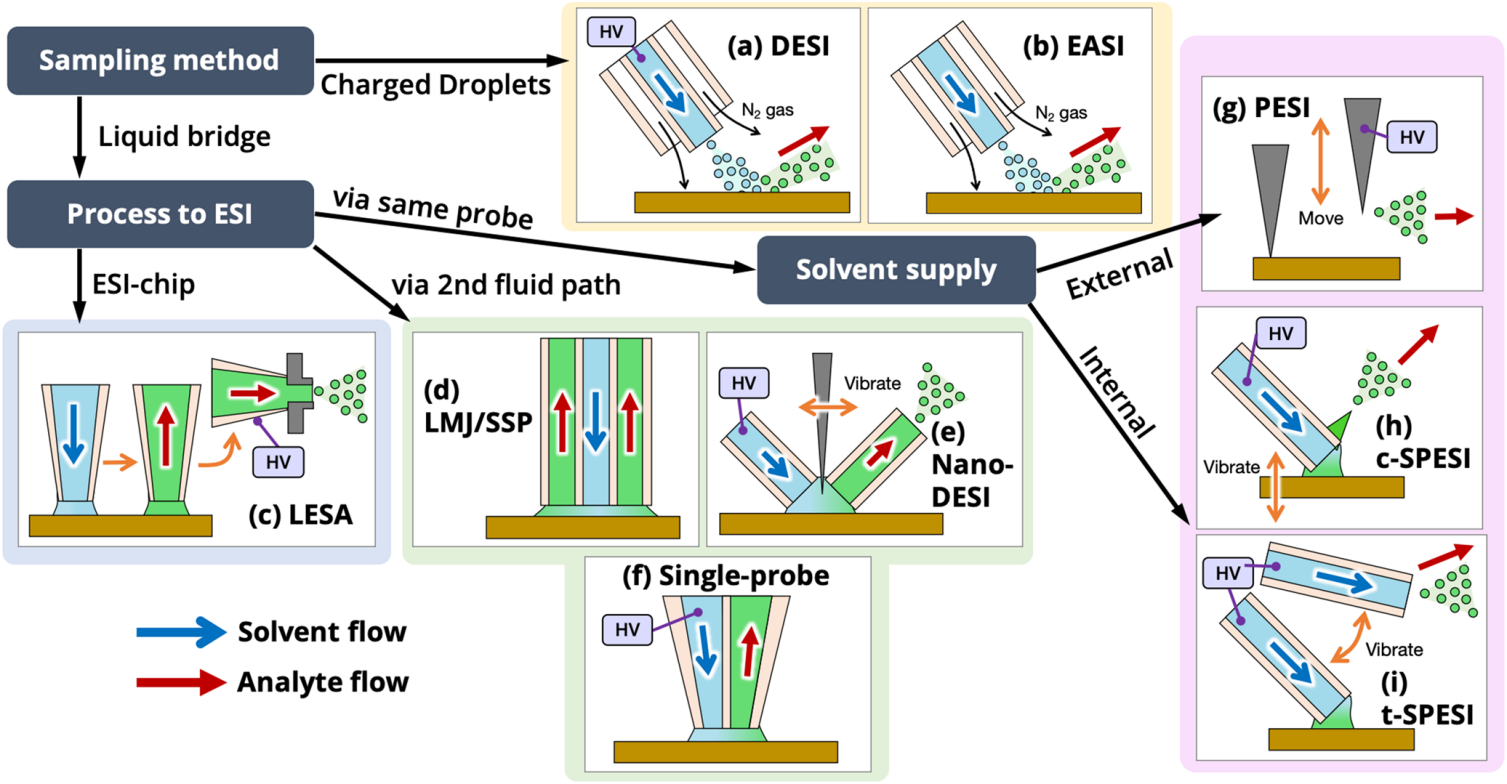
Fig. 5. Classification of DLEIs described in the present review.

DLEIs can be described using a classification based on three levels of categories, as shown in Fig. 5. There are two methodologies for sample extraction: using charged droplets and using a liquid bridge. The former includes desorption electrospray ionization (DESI, Fig. 5(a)) and easy ambient sonic spray ionization (EASI, Fig. 5(b)), whereas the others are included in the latter group.

There are also two methodologies by which the sample solution extracted by the liquid bridge is subjected to ESI. The first group includes methods that utilize a different flow path for ESI. The method utilizing a dedicated ESI chip is named liquid extraction surface analysis (LESA, Fig. 5(c)). Methods that use a second flow path to transport the extracted solution include liquid microjunction surface sampling probe (LMJ/SSP, Fig. 5(d)), nanospray desorption electrospray ionization (nano-DESI, Fig. 5(e)), and single-probe (Fig. 5(f)).

The second group includes methods for liquid bridge formation and extraction solution transfer by the same probe. They can be further distinguished by the solvent supply method. Probe electrospray ionization (PESI, Fig. 5(g)) is a method in which the solvent is supplied from the outside of the probe, and scanning probe electrospray ionization (SPESI, Fig. 5(h) and (i)) is a method in which the solvent is supplied from inside the probe. These two techniques were invented in Japan.

The different methods for supplying solvent and transferring the extracted solution for ESI result in differences in the area of extraction, the time interval between extraction and ionization, and the ability to perform measurements in response to the unevenness of the sample and/or the tilt of the substrate. The techniques involved for each method and their application to biological samples are described below.

### 3.1 DESI

DESI^57)^ (Fig. 5(a)) is the most widely studied and commercially available DLEI method. The ion source consists of a coaxial capillary. Charged solvent is supplied from the inner capillary and high-pressure nebulizing gas is supplied from the outer capillary. In an optimized DESI ion source,^58)^ the nebulizing gas is 4–4.5 and 7 bar for solvent flow rates of 0.5 and 1.5 μL/min, respectively. A stream of charged solvent droplets (velocity: 120 m/s, average particle size: 2–4 μm) is generated from the end of the ion source^59)^ and collides with the sample surface to desorb and ionize the sample components.

As a model of desorption and ionization, a thin film of charged solvent is formed on the sample surface to extract the sample components locally. The charged droplets continuously colliding onto the thin film cause the desorption of secondary droplets from the surface. The desorbed droplets are believed to produce gas-phase ions by the same mechanism as in ESI^60)^ and have an angular distribution, and thus the angle and position of the ion source and inlet must be adjusted appropriately.^61)^ Given that the charged droplets spread on the sample surface, the general size of the extraction region is about 100 μm in diameter.

There are a number of reports on the MSI of biological tissues using DESI, many of which are related to the visualization of localized chemical components in diseased tissues and of drugs in biological tissues. Representative reports such as mouse whole body tissue sections,^62)^ human prostate cancer,^63,64)^ human astrocytoma,^65)^ human renal cell carcinoma,^66,67)^ human seminoma,^68)^ human brain tumor,^69)^ human colorectal adnocarcinoma,^70)^ mouse whole body tissue,^71)^ human lymph node metastasis,^72,73)^ human breast cancer,^74,75)^ human lymphoma,^76)^ human skin,^77)^ human gastric cancer,^78)^ human meningioma,^79)^ lipid profile of human brain,^80)^ human ovarian cancer,^81,82)^ human pancreatic cancer,^83)^ human thyroid tumor,^84)^ human lymph node breast cancer and thyroid cancer,^85)^ intraoperative evaluation of brain tumors,^86,87)^ human oral squamous cell carcinoma of the tongue,^88)^ human skin lesions^89)^ and ovaries of cows, sows, and mice.^90)^

**Figure 6:**
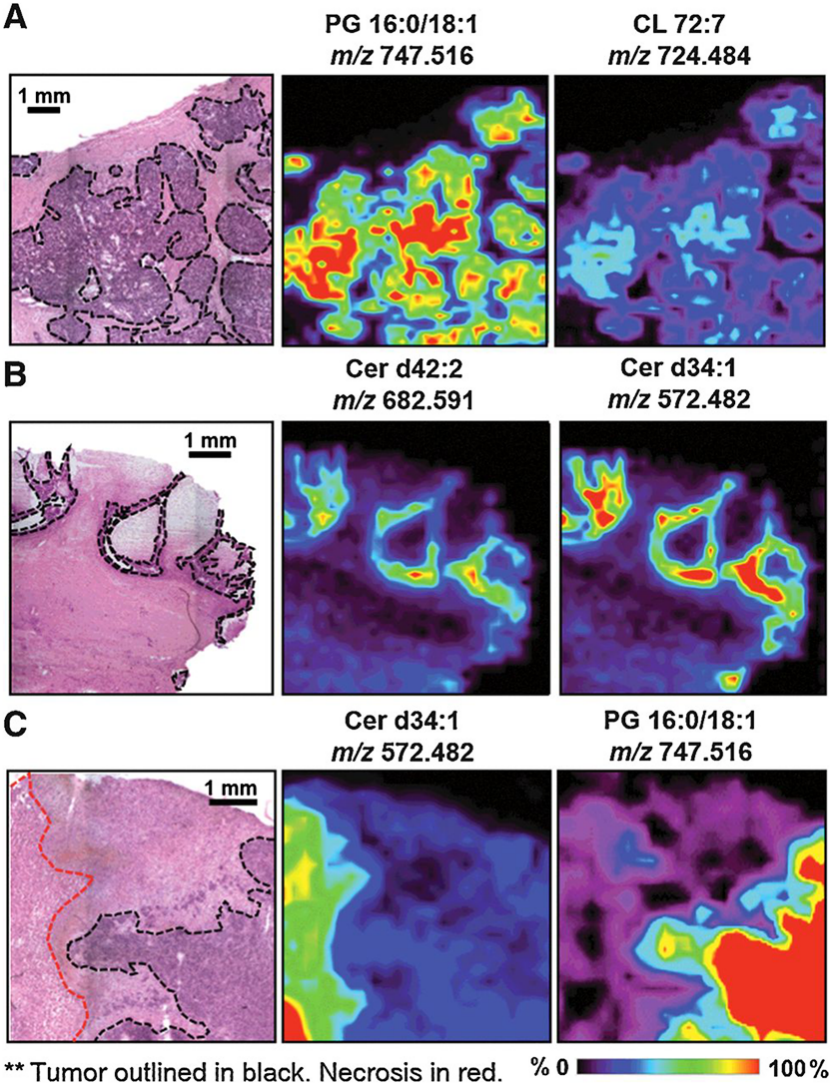
Fig. 6. Mass spectrometric imaging of human ovarian cancer tissue using DESI, showing lipids localized in the region identified as tumor by HE staining. Reproduced with permission from Ref. 82. Copyright (2017) American Association for Cancer Research.

In the MSI of diseased tissues, examples of which are shown in Fig. 6, components localized to the diseased tissues can be visualized by comparing them with hematoxylin–eosin (HE) stained images, a standard method for pathological diagnosis. Multivariate analysis of highly localized component groups can also be used to obtain features for classifying diseases.^82)^ In general, MSI is effective in capturing the distribution of disease-related components in tissues but requires a long measurement time. An application of DESI for rapid intraoperative diagnosis utilizes smear analysis (a sample of tissue fragments removed during surgery and spread on a glass slide),^86,87,91)^ but increased measurement throughput comes at the expense of sample location information.

### 3.2 EASI

**Figure 7:**
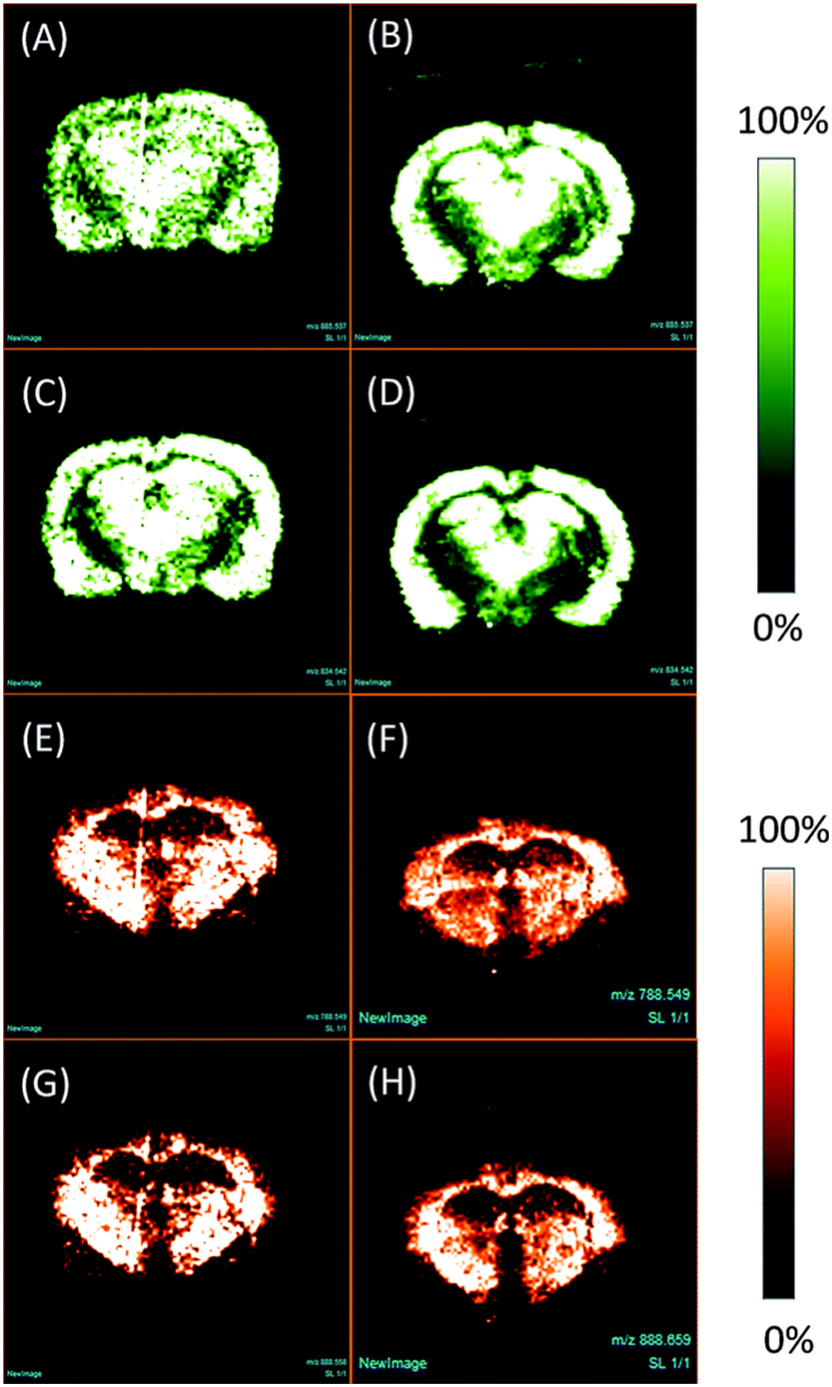
Fig. 7. Imaging results of rat brain tissue using EASI. (A), (C), (E), (G) were obtained using EASI and (B), (D), (F), (H) were obtained using DESI. Reproduced with permission from Ref. 95. Copyright (2017) Royal Society of Chemistry.

EASI^92)^ (Fig. 5(b)) is similar to DESI in the configuration of the ion source. The main difference is that it does not apply high voltage to the solvent but uses nitrogen or air flow to generate charged droplets. The sonic spray^93,94)^ charges the droplets by creating a non-uniform charge in the solvent. The charged droplets are sprayed onto the sample surface to desorb and ionize the sample components. High voltage is not used in EASI, and it is reported to provide milder atmospheric sampling ionization compared with DESI. A comparison of MSI with DESI and EASI in rat brain tissue showed that EASI provided similar or slightly higher sensitivity compared with DESI in detecting lipids^95)^ (Fig. 7). EASI is thus the technique of choice when it is necessary to avoid electrochemical effects on the sample caused by the application of high voltage.

### 3.3 LESA

LESA^96)^ (Fig. 5(c)) supplies solvent to the sample surface *via* a pipette. A liquid bridge is formed between the pipette and the sample and held for a certain period of time before the extract is sucked up into the pipette. The pipette and the ESI tip are changed for each measurement and thus there is no carry-over of sample.

**Figure 8:**
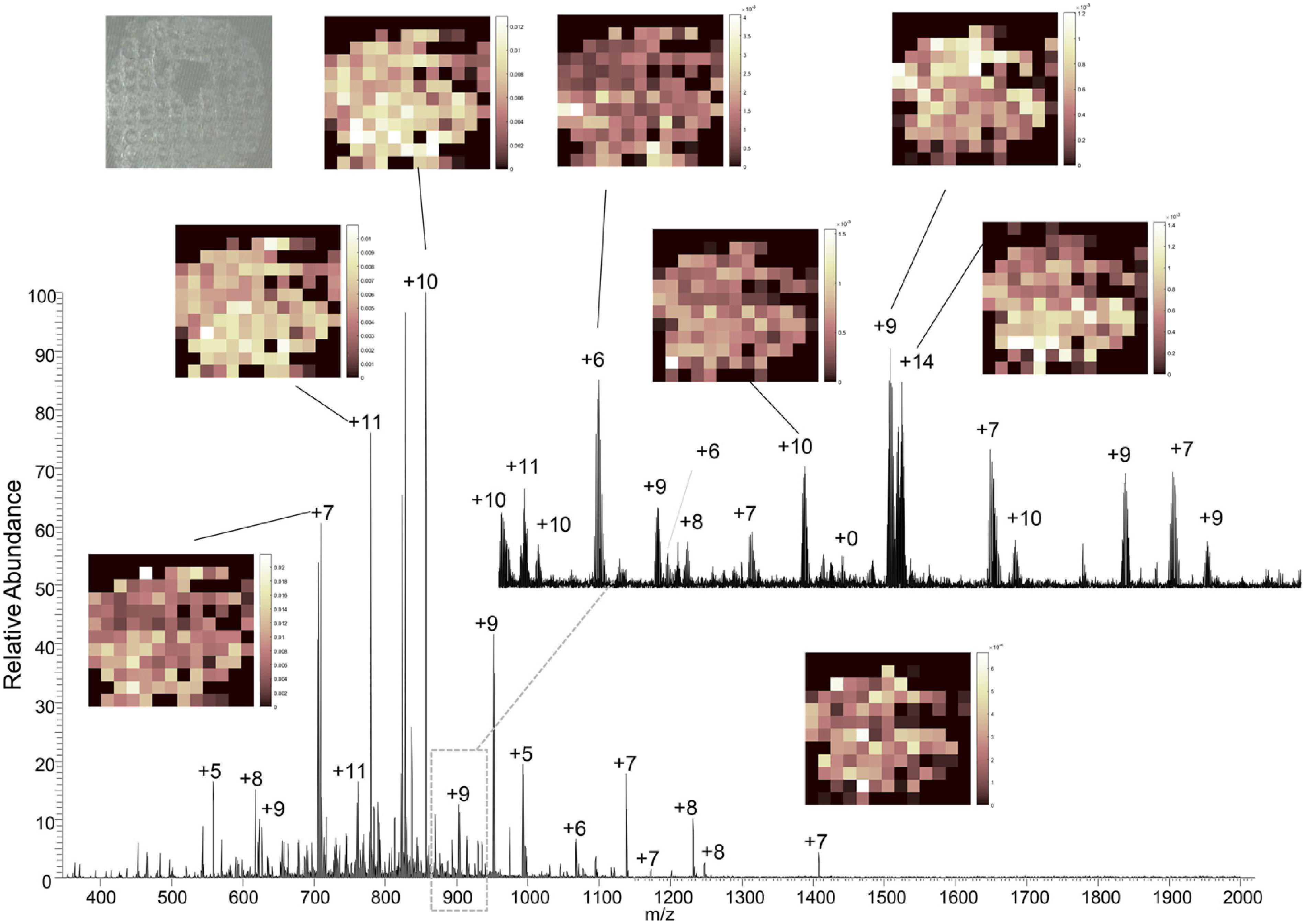
Fig. 8. Mass spectrometric imaging of proteins in mouse testis tissue using LESA. Reproduced with permission from Ref. 102. Copyright (2020) American Chemical Society. https://pubs.acs.org/doi/10.1021/acs.analchem.9b05124

MSI can be performed by changing the measurement position on the sample surface, although measurements take longer. Depending on the aperture size of the pipette, the size of the extraction area is about 1 mm. LESA instrumentation is commercially available, and many tissue measurements using LESA have been reported. For example, LESA has been used to identify drugs and metabolites in rat whole-body tissues,^97)^ proteins in tissue sections,^98–102)^ proteins in bacterial colonies,^103,104)^ drugs in rat brain,^105)^ liver,^106)^ kidney^107)^ and dried blood,^108)^ and to identify lipids in human non-alcoholic fatty liver disease^109)^ and in breast cancer cells.^110)^ Recently reported results are shown in Fig. 8, where the distribution of proteins (975 types) in mouse testicular tissue was measured using a combined LESA and high field asymmetric waveform ion mobility spectrometry (FAIMS) measurement system.^102)^

### 3.4 LMJ/SSP

The LMJ/SSP technique^111,112)^ (Fig. 5(d)) utilizes a capillary with a coaxial tube structure. The solvent is supplied to the sample from the inner capillary and the extractant is drawn up from the outer capillary. When the end of the capillary is brought into close proximity with the sample surface, a liquid bridge of solvent is formed between the two, and the sample components are extracted. The extracted solution is transported to the ESI ion source for ionization. The size of the liquid bridge can be controlled by adjusting the solvent flow rate and the suction rate of the extractant.^113)^ Due to the thickness of the coaxial capillary, the size of the extraction region is approximately 600 μm. The shape of the liquid bridge can be controlled using a technique to control the distance between the capillary and the sample surface for MSI.^114)^ Biological tissue measurements using LMJ/SSP include fresh mouse brain tissues,^115,116)^ drugs in tissues,^117–119)^ and proteins in sheep blood spots.^120)^

**Figure 9:**
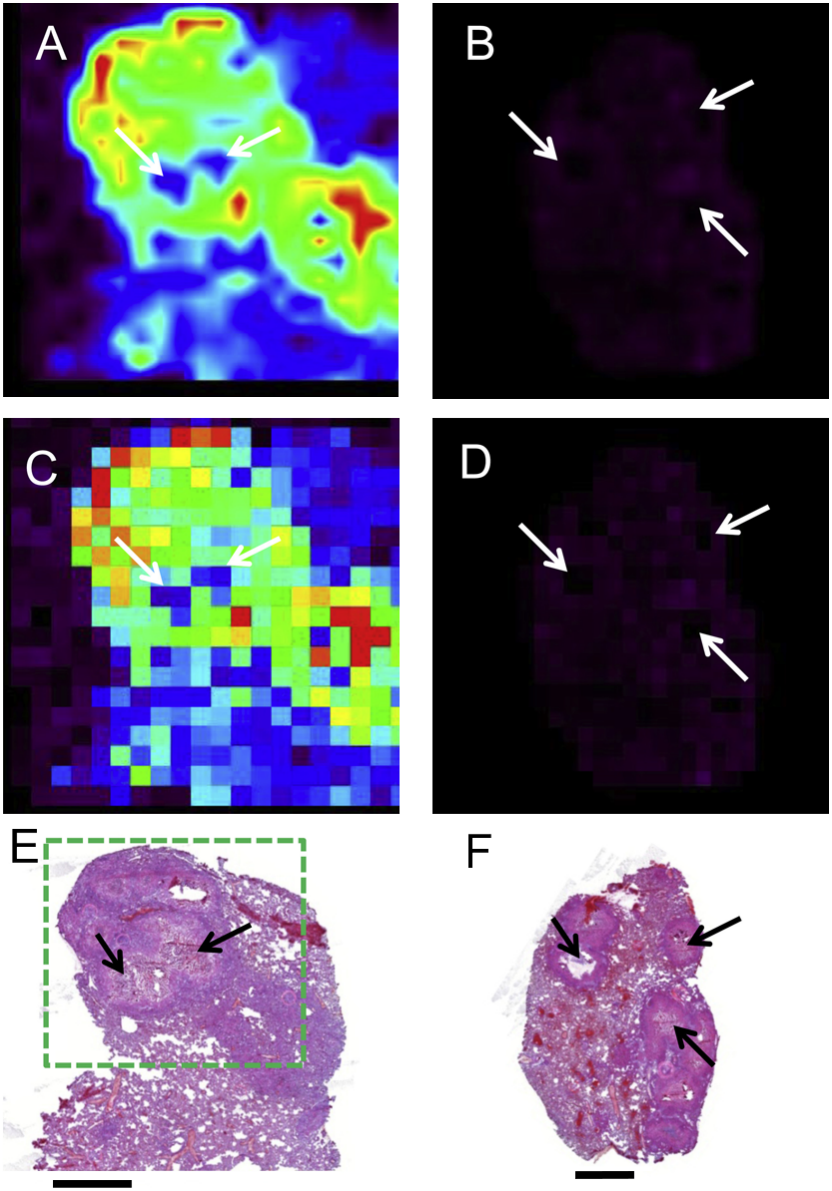
Fig. 9. Imaging results of drug distribution in rat tissues using LMJ/SSP. Reproduced with permission from Ref. 119. Copyright (2015) Elsevier.

Figure 9 shows the MSI results of drug distribution in rabbit tissues using a commercially available device. Imaging with a spatial resolution of 630 μm shows the temporal changes in drug distribution after administration (Fig. 9(A) shows the results following a smoothing process and Fig. 9(C) shows the raw data).^119)^ The authors of that study also compared LMJ/SSP with MALDI. Although the spatial resolution of LMJ/SSP was lower than that of MALDI, LMJ/SSP requires much shorter measurement time, no sample preparation, and components destabilized by laser light or vacuum can be measured. A measurement system combining LMJ/SSP and ultrahigh- or high-performance liquid chromatography (UPLC, HPLC) showed high identification and quantitative performance.^116,120)^

### 3.5 Nano-DESI

In nano-DESI^121)^ (Fig. 5(e)), two capillaries are arranged in a V-shape and the charged solvent is supplied to the sample surface through the first capillary to form a liquid bridge between the sample surface and the two capillaries. The sample components are dissolved in the liquid bridge, and the second capillary guides the extracted solution for ESI in front of the inlet. Nano-DESI utilize a smaller volume liquid bridge compared with other methods, and thus provides high sensitivity measurement^122)^ and high spatial resolution imaging. MSI of biological tissues with a spatial resolution of 12 μm has been reported.^123)^

Another advantage of nano-DESI is the development of technology to perform DLEI that follows the surface shape of the sample. The actual sample is uneven and tilted, requiring a technique to perform DLEI without being affected by these factors to obtain reproducible measurements and high spatial resolution imaging. To achieve this, a third shear force probe is positioned between the two capillaries to monitor the liquid bridge. Automatic control of the height of the sample results in the vibration amplitude of the third probe remaining constant, allowing high spatial resolution MSI.^124,125)^

**Figure 10:**
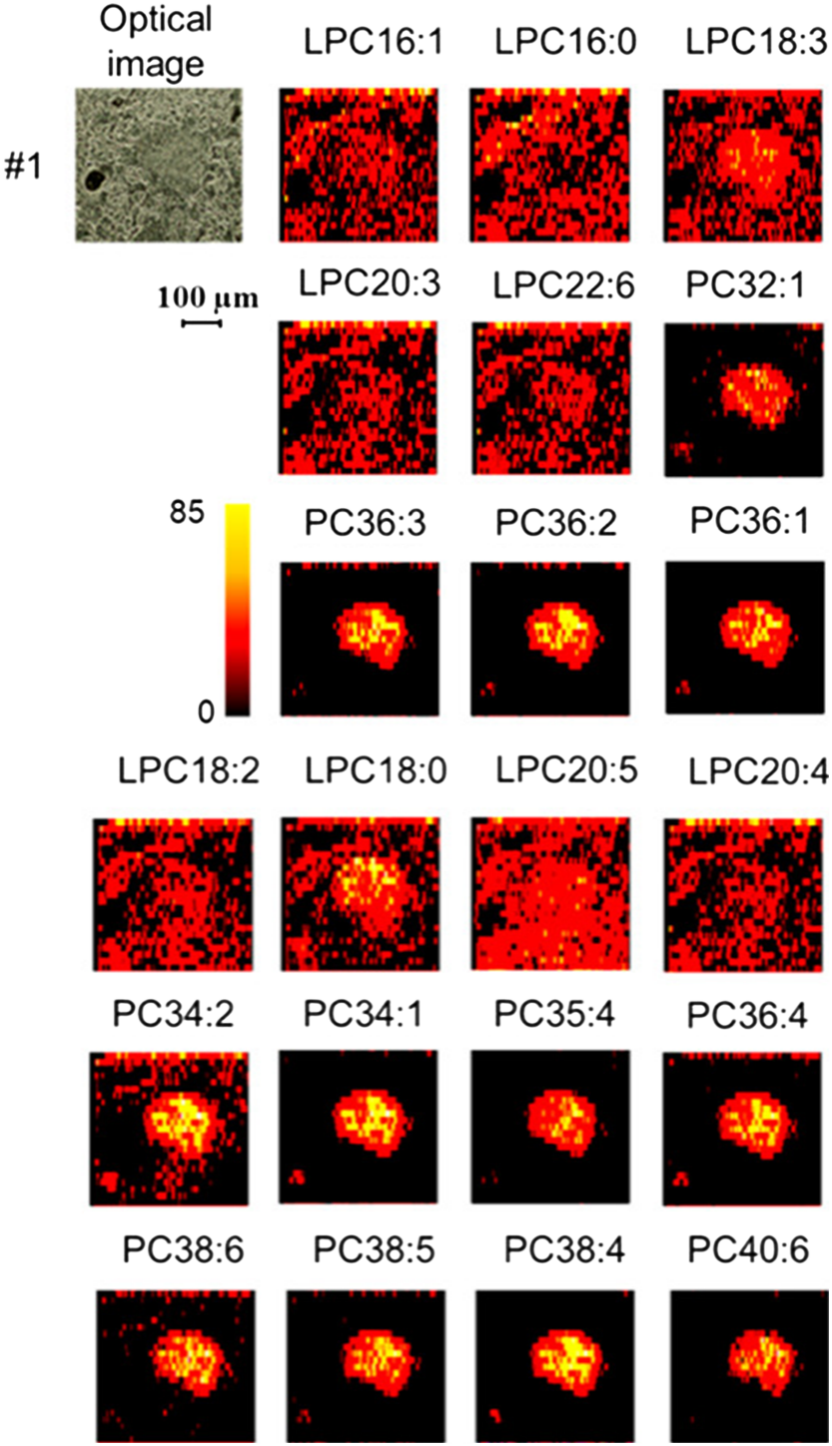
Fig. 10. High-resolution mass spectrometry imaging of mouse pancreas tissue using nano-DESI. Reproduced with permission from Ref. 123. Copyright (2018) American Chemical Society.

Tissue MSI using nano-DESI has been reported for mouse pancreatic islets,^123)^ murine gastrocnemius muscle,^126)^ proteins in mouse brain,^127)^ and prostaglandins in mouse uterus.^122)^ Figure 10 shows the results of MSI of mouse pancreatic tissue using a feedback control system. The distribution of metabolites and lipids in pancreatic islets several hundred μm in size could be visualized with a spatial resolution of 11 μm.^123)^

### 3.6 Single probe

Single-probe^128)^ (Fig. 5(f)) uses a quartz probe with two side-by-side flow channels. Similar to LMJ/SSP and nano-DESI, a solvent with high voltage is supplied to the sample surface *via* a flow channel, and the components are extracted in the liquid bridge. The extracted solution is guided to the second flow channel and transported to the ESI source. Like nano-DESI, high spatial resolution imaging is possible; for example, the spatial resolution of MSI of mouse tissue is about 8–17 μm.^129)^ The probe tip is small enough to be inserted into a cell, allowing single-cell analysis.^128,130)^ However, there is no mechanism for controlling the distance between the probe and the sample, unlike nano-DESI, and so it is difficult to follow the unevenness of the sample.

### 3.7 PESI/SF-PESI

**Figure 11:**
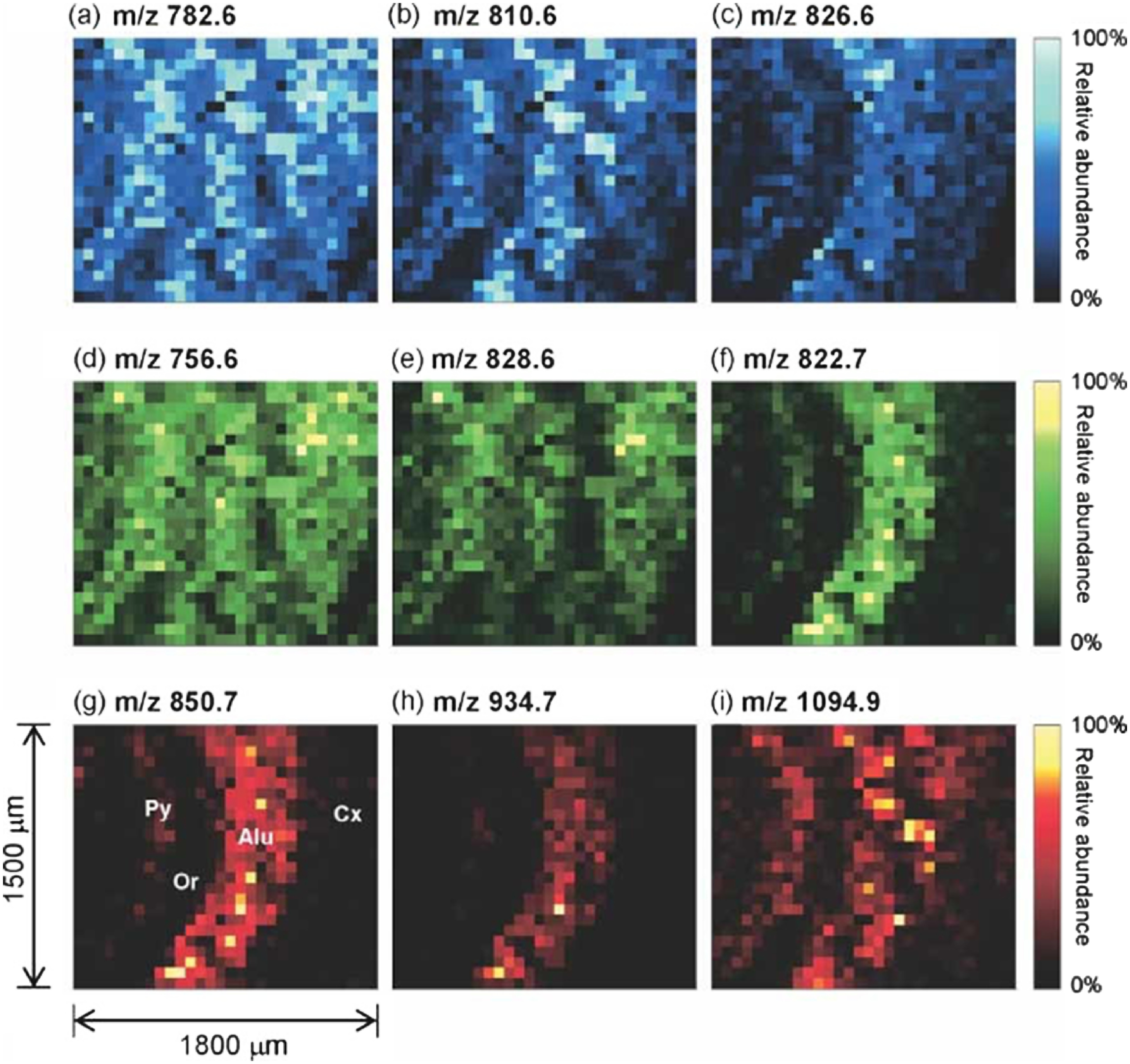
Fig. 11. Mass spectrometric imaging of mouse brain tissue using PESI. Reproduced with permission from Ref. 135. Copyright (2009) John Wiley and Sons.

In PESI^131)^ (Fig. 5(g)), the sample solution is in contact with a metal needle and ionized by ESI by applying a high voltage. The vertical motion of the probe (3 Hz) and the timing of high voltage application to the probe are controlled.^132)^ A small volume of sample solution, ranging from a few pL to several hundred fL, is ionized. Of all the methods presented in this review, PESI can perform extractive ionization on the smallest amount of sample solution. In addition, sequential ionization, which will be discussed later, can be used to reduce ion suppression effects.^133)^ PESI has been used to identify human cancer tissue,^134)^ and MSI imaging of mouse brain tissue with a spatial resolution of 60 μm has been reported^135)^ (Fig. 11).

Sheath flow PESI (SF-PESI) is a derivative technology of PESI.^136)^ A metal needle is inserted into a gel loading tip containing solvent so that the needle tip protrudes slightly. Sample extraction is performed by touching the needle to the sample, and ESI is performed by applying a high voltage to the needle.

### 3.8 SPESI

SPESI allows fast extraction and ionization with a single capillary probe. Two different modes of operation have been proposed.^137)^

In contact-mode SPESI (c-SPESI, Fig. 5(h)), a high voltage is applied to a solvent flowing inside a silica capillary probe. The probe end is in contact with the sample surface and the backside of the sample is subjected to ultrasonic vibration. A liquid bridge is formed between the probe end and the sample, and the sample components are extracted. The extracted liquid moves in the direction of the electric field and ESI is performed.

In tapping-mode SPESI (t-SPESI, Fig. 5(i)), the probe is vibrated, and the sample and probe end are in intermittent contact. A liquid bridge and electrospray are formed alternately in a short time (the two processes can be separated spatiotemporally). The probe can be vibrated at a resonant frequency using a piezoelectric actuator^138)^ or at an appropriate frequency using a mechanical relay.^139)^

The flow rate of the solvent is about several hundred nL/min, and the vibration frequency of the probe can be varied in the range of 200–1000 Hz by adjusting the length between the fixed end and the probe end. The characteristics of t-SPESI are that the volume of the extracted solvent can be reduced by using a vibrating probe, and that the extracted components can be rapidly ionized. Thus, higher ionization efficiency can be expected due to the solvent volume effect in ESI described below. Using a probe with a fine tip shape provides extraction areas of 5–6 μm.^140)^

**Figure 12:**
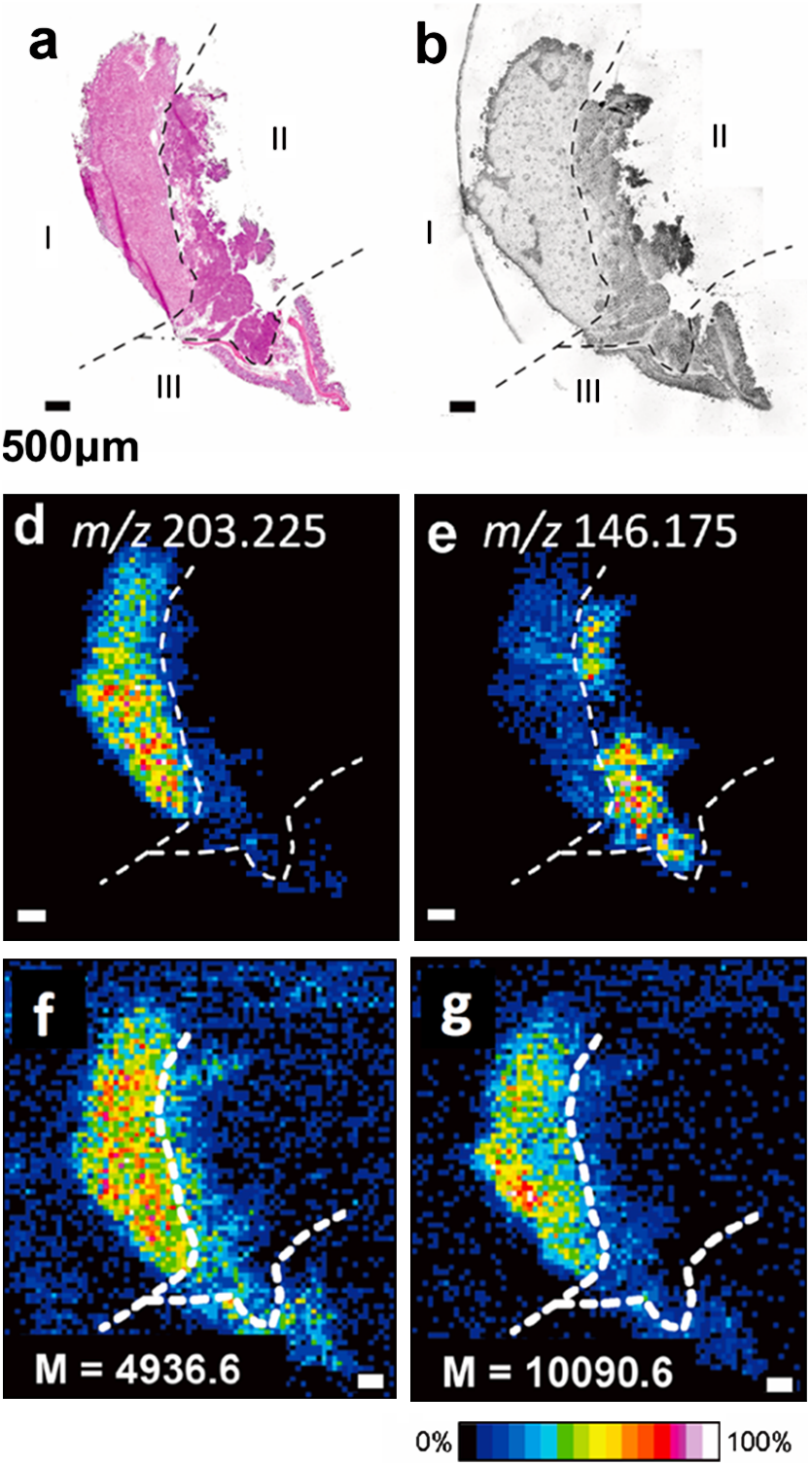
Fig. 12. Mass spectrometric imaging of mouse pancreatic cancer tissue using t-SPESI. Reproduced with permission from Ref. 141. Copyright (2015) John Wiley and Sons.

MSI by t-SPESI has been applied to mouse brain^138)^ and mouse pancreatic cancer samples.^141)^ Figure 12 shows the results of MSI of mouse pancreatic cancer tissue, with simultaneous measurement of low-molecular-weight metabolites and the abundance of proteins localized in the cancerous region (I) and normal region (II).

A method has also been developed to measure probe displacement with a laser beam. Using this method, the formation and breakage of liquid bridges during a single extraction and ionization process by c-SPESI was observed to vary depending on the solvent type.^142)^ In addition, a feedback control system to maintain a constant vibration amplitude of the probe in t-SPESI by controlling the sample height is important to stabilize measurements.^143)^

A comparison of the spatial resolution, solvent supply method, availability of commercial equipment, features, and compatible solvent species of the above DLEIs is summarized in Table 1.

**Table 1:** Table 1. Comparison of DLEIs.

Acronym	Technical name	Sampling resolution (μm)	Method for supplying solvent	Timing of sampling and ionization	Commercialized	Feature	Solvents used for biological analysis
DESI	Desorption electrospray ionization	30–200	Charged solvent droplet with nebulizing gas	Simultaneous	Yes	Rapid sampling and ionization Applicable to Reactive DESI	*N*,*N*-Dimethylformamide (DMF)/EtOH (1 : 1) DMF/Acetonitrile (ACN) (1 : 1) ACN/EtOH (1 : 1) MeOH/CHCl_3_ (1 : 1) ACN/CHCl_3_ (1 : 1)
EASI	Easy ambient sonic spray ionization	30–200	Charged solvent droplet with nebulizing gas	Simultaneous	No	Rapid sampling and ionization	MeOH
LESA	Liquid extraction surface analysis	1000	Pipette	Separated	Yes	Large area analysis	200 mM ammonium acetate (5% MeOH) ACN : 0.1% TFA (8 : 2), MeOH/H_2_O/formic acid (FA) (69.5 : 29.5 : 1) ACN/H_2_O/FA (39.5 : 59.5 : 1) 10 mM ammonium acetate (5% MeOH or 0.125% ﻿tetraethylene glycol monooctyl ether (C8E4))
LMJ/SSP	Liquid microjunction surface sampling probe	600	Coaxial capillary	Separated	Yes	Large area analysis	MeOH/CHCl_3_ (3 : 1) MeOH (0.1% FA) MeOH/H_2_O (1 : 1) MeOH/H_2_O (1 : 1, 0.1% FA) ACN/H_2_O/FA (10 : 90 : 0.1) MeOH/H_2_O (9 : 1) MeOH/isopropanol/1,1,1,3,3,3-hexafluoro-2-propanol (8 : 1 : 1)
Nano-DESI	Nanospray desorption electrospray ionization	12–150	V-shape capillary (separated)	Separated	No	Feedback control system High resolution imaging Applicable to Reactive nano-DESI	MeOH/H_2_O (9 : 1) ACN/MeOH (0.1% FA) with Ag ion MeOH/dichloromethane (6 : 4), MeOH/ACN/toluene (5 : 3.5 : 1.5) ACN/H_2_O (65 : 35, 0.2% FA)
Single-probe		8.5	V-shape capillary (integrated)	Separated	No	High resolution imaging	MeOH/H_2_O (9 : 1) ACN
PESI	Probe electrospray ionization	60	Externally	Separated	Yes	Sequential ESI	MeOH/H_2_O/acetic acid (50 : 50 : 1) MeOH/H_2_O (1 : 1) EtOH/H_2_O (1 : 1) Isopropanol/H_2_O (1 : 1) *n*-Propanol
c-SPESI	Contact-mode Scanning probe electrospray ionization	100	Single capillary	Simultaneous	No	Rapid sampling and ionization	MeOH/H_2_O (1 : 1, 0.2% FA)
t-SPESI	Tapping-mode Scanning probe electrospray ionization	5–100	Single capillary	Separated	No	Rapid sampling and ionization High resolution imaging	DMF/EtOH (1 : 1) MeOH/H_2_O (0.2% FA or 0.1% FA)

## 4. DLEI METHODS FOR BIOLOGICAL SPECIMENS

### 4.1 Solvent selection

In DLEI, the type of solvent is believed to affect the efficiency and selectivity of extraction and ionization. This section describes the solvents used to date for the analysis of biological tissues.

HE staining and other staining techniques have been used in pathology to observe the morphology and chemical state of biological tissues to diagnose diseases. In order to study the relationship between the distribution of chemical components in a tissue and the disease state, it is standard to compare the results of MSI of unstained tissue with optical images of stained tissue.

The relationship between the type of solvent and the change in shape of the tissue due to the DESI extraction process has been reported.^144)^ Solvents that do not affect sample morphology include *N*,*N*-dimethylformamide (DMF)/EtOH (1 : 1), DMF/acetonitrile (ACN) (1 : 1), ACN/EtOH (1 : 1), MeOH/CHCl_3_ (1 : 1) and ACN/CHCl_3_ (1 : 1), with the first two mixtures providing high detection sensitivity. All these solvents are appropriate for staining tissue sections after MSI for pathological analysis. However, MeOH/H_2_O (1 : 1), EtOH/H_2_O (1 : 1) and ACN/H_2_O (1 : 1) were shown to deform tissue morphology, likely due to a thin film of solvent with high surface tension forming on the surface of the tissue, which is removed by the high-speed nebulizing gas, simultaneously peeling off the tissue.

In LESA, 200 mM ammonium acetate solution (5% MeOH),^7)^ ACN : 0.1% TFA solution (8 : 2),^98)^ and MeOH/H_2_O/formic acid (FA) (69.5 : 29.5 : 1) mixtures were used to measure mouse liver tissue, an ACN/H_2_O/FA (39.5 : 59.5 : 1) mixture was used to measure mouse brain tissue,^145)^ an ACN/H_2_O (40 : 60, 1% FA) mixture was used to measure mouse tissue proteins,^99)^ and 10 mM ammonium acetate solution (5% methanol or 0.125% tetraethylene glycol monooctyl ether (C8E4)) was used to measure rat tissue proteins.^101)^

In LMJ/SSP, a MeOH/CHCl_3_ (3 : 1) mixture was used to measure metabolites in mouse brain,^115)^ MeOH (0.1% FA) was used to measure drugs in bovine, porcine and equine tissues,^117)^ MeOH/H_2_O (1 : 1, 0.1% FA) was used to measure metabolites and drugs in rat liver,^118)^ and ACN/H_2_O/FA (10 : 90 : 0.1) was used to measure drugs in rat liver.^120)^ MeOH/H_2_O (9 : 1) and MeOH/isopropanol/1,1,1,3,3-hexafluoro-2-propanol (8 : 1 : 1) mixed solvent were used for lipid measurements in rat brain tissue.^116)^

In nano-DESI, MeOH/H_2_O (9 : 1) mixture is the preferred solvent.^123,146–148)^ ACN/toluene/MeOH (7 : 3 : 10) was used to measure lipids,^149)^ and ACN/MeOH (0.1% FA) with silver ions was used to measure prostaglandins.^122)^ MeOH/dichloromethane (6 : 4) and MeOH/ACN/toluene (5 : 3.5 : 1.5) mixtures were used for triglyceride measurements^126)^ and protein in tissues were measured using 65% ACN (0.2% FA, aqueous solution).^127)^

In PESI, MeOH/H_2_O/acetic acid (50 : 50 : 1), MeOH/H_2_O (1 : 1), EtOH/H_2_O (1 : 1), isopropanol/H_2_O (1 : 1) and *n*-propanol were used to measure human renal cell carcinoma.^150)^

In t-SPESI, a DMF/EtOH (1 : 1) mixture was used for lipid measurements of mouse brain tissue^138)^ and MeOH/H_2_O (0.2% FA or 0.1% FA) was used for measurements of mouse pancreas tissue.^137,141)^ Solvent selectivity and optimization will be necessary for each specific technique in the future.

### 4.2 Sample preparation

The detection of proteins and peptides in biological tissue sections using DLEI requires a pretreatment step to remove abundant lipids because lipids are preferentially ionized over proteins.

For DESI measurements, tissue sections (mouse kidney and brain, and human ovary and breast) were washed twice with EtOH and CHCl_3_ for 10 s.^151)^ For LESA, rat brain tissue sections were immersed sequentially in 70% EtOH, 95% EtOH, and CHCl_3_ for 30 s, and dried in a vacuum desiccator.^98)^ The surfaces of mouse liver and brain tissue sections were washed with EtOH/H_2_O (80 : 20), followed by extractive ionization with the measurement solvent.^145)^ In other case, a mouse brain section was treated by washing with 70^102)^ or 80% EtOH^99)^ for 10 s. For nano-DESI measurements, mouse brain tissue sections were washed twice with CHCl_3_.^127)^ In contrast, t-SPESI detected proteins in mouse pancreatic cancer tissue without any pretreatment,^141)^ suggesting that different DLEI methods may ionize proteins without tissue pretreatment.

## 5. ION SUPPRESSION OF MULTI-COMPONENT SAMPLES AND COUNTERMEASURES

### 5.1 Electrospray ionization

The factors that affect ESI can be classified into measurement system variables, compound variables, and method variables.^152)^ Measurement system variables include the electric field, emitter diameter, voltage applied to the emitter, distance to the counter electrode, heat capacity and solvent saturation level of ambient gas. The compound variables include surface activity, proton affinity, acid dissociation constant (p*K*_a_), and solvation energy. The method variables include flow rate, electrolyte concentration, pH,^153)^ and physicochemical properties of the solvent such as boiling point and surface tension. The presence of non-volatile solutes such as salt in the sample solution increases the boiling point and surface tension of the solution, decreases the formation rate of micro-charged droplets generated from larger micro-charged droplets, and suppresses ionization.^154)^ Furthermore, highly polar molecules are reported to be susceptible to ion supression.^155)^

In the direct analysis of biological samples (*e.g.*, blood, urine, extracts of biological tissues) using liquid chromatography-mass spectrometry (LC-MS), ionization suppression is minimized by sample purification and separation.^156)^ However, it is difficult to completely avoid ionization suppression in the direct analysis of biological samples because multiple components are extracted and ionized in a short time.

Ion suppression without pretreatment can be reduced by reducing the flow rate of the sample solution. Ionization at a lower flow rate than is used for ESI is called nano-ESI. In nano-ESI, fine capillaries with outer diameters on the order of μm^157)^ to nm^8,158)^ are used to pump the solution at low flow rates of tens of nL/min^159)^ to pL/min.^160)^ These low flow rates are important to reduce the size of the initial charged droplets generated from the Taylor cone. The smaller the initial droplet volume, the fewer the number of Coulomb fissions required before the gas-phase ions are released, thus improving ionization efficiency and reducing ion suppression by buffers, detergents, and salts.^8,161–163)^

Given that the size of the charged droplet is correlated with the lifetime of the droplet, the capillaries used in nano-ESI have the advantage of preventing protein denaturation. For example, the ionization of β-lactoglobulin using capillaries with open apertures of 1.7–4.4 μm resulted in droplet lifetimes of more than 10 μs and denatured proteins were measured. In contrast, a capillary with an aperture of 317 nm was reported to reduce the droplet lifetime to about 1 μs, thus reducing the amount of denatured protein.^164)^

### 5.2 Direct liquid extraction ionization

A study comparing ion suppression in ESI and DESI^165)^ showed that DESI was less likely to cause ion suppression compared with ESI when the molecules in the mixture solution (drug molecules such as atenolol and thioconazole, and ammonium salts with different alkyl chains) were measured under similar conditions. The ionization mechanism in DESI is different from that of ESI. In ESI, the larger the emitter aperture and flow rate, the larger the droplet size and the lower the ionization efficiency. In DESI, large droplets stay on the sample surface for a long time and contribute to the extraction of sample components, while small droplets are desorbed from the sample and contribute to ionization. The difference in the amount of small droplets is believed to cause the observed difference in ionization efficiency.

In a comparison of lipid measurements in mouse lung tissue using nano-DESI and liquid chromatography-tandem mass spectrometry (LC-MS/MS),^147)^ the number of lipids measured by nano-DESI was about 50% of that measured by LC-MS/MS in extracts prepared using the Forch method. Although direct comparison is difficult due to the different extraction processes used for the LC-MS/MS samples, ion suppression by nano-DESI was higher because multiple lipids are ionized simultaneously and the area of the sample extracted is 100 μm^2^, which reduces the number of molecules. In contrast, the merits of nano-DESI include the ability to measure small molecules such as monoglycerides and metabolites that cannot be measured by LC-MS/MS, and visualization of the distribution of lipid components, which is complementary to LC-MS/MS.

With PESI, the volume of the sample solution can be reduced to the order of pL and sequential ionization is possible, thus reducing ion suppression. In one study, the volume of the sample solution attached to the metal needle after contacting the needle with the sample was 0.35±0.09 to 5.69±1.70 pL, depending on the analyte in the solvent.^132)^ Importantly, when ESI is generated by applying a voltage to the mixture solution attached to the needle, the molecules are sequentially ionized in a few tens of seconds, depending on their surface activity.^133)^ Because this sequential ESI ionizes the mixture while separating the components on the probe, it helps reduce ionization suppression by surfactants and salts in the solution and thus is useful for the clinical analysis of biological tissues^150)^ and serum.^166)^

Internal standards have been used for the quantitative analysis of biological tissues for nano-DESI to quantitatively evaluate the concentration of an analyte based on the intensity of an internal standard in a solvent. The use of internal standards has allowed measurements of nicotine,^167)^ phospholipids,^168)^ and neurotransmitters (acetylcholine, γ-aminobutyric acid, and glutamate)^169)^ in brain tissue.

## 6. UTILIZATION OF CHEMICAL REACTIONS

Apart from using DLEI to measure a wide range of chemical components of a sample, the highly sensitive and selective ionization of specific chemical components achieved using DLEI is important for measuring trace amounts of biomarkers. Specific chemical reactions in charged droplets and examples of derivatization for DLEI are described below.

### 6.1 Derivatization for mass spectrometry

Derivatization reagents for MS have been developed mainly to change the physical and chemical properties of the molecules to be measured, to determine their structures, to shift their masses, and to improve detection sensitivity.^170–176)^ In addition, techniques for combining derivatization with LC-MS have been developed since the 1980s to aid soft ionization methodologies.^177–179)^

Girard’s derivatization is utilized in ESI-MS. Girard’s reagent reacts with aldehydes and ketones under mild acidic conditions to form hydrazone derivatives^180)^; 61 years after it was first reported, Girard’s reagent was used to derivatize steroid esters for ESI-MS analysis. The presence of a quaternary amine in the derivative increased ionization efficiency and produced a characteristic fragmentation pattern of [M−59]^+^ and [M−87]^+^ (M^+^: molecular ion of the steroid derivative) in tandem mass spectrometry measurements.^181)^ Girard’s derivatization has been used to quantitatively evaluate ketosteroids and the derivatization of fatty aldehydes and ketosteroids.^182)^

**Figure 13:**
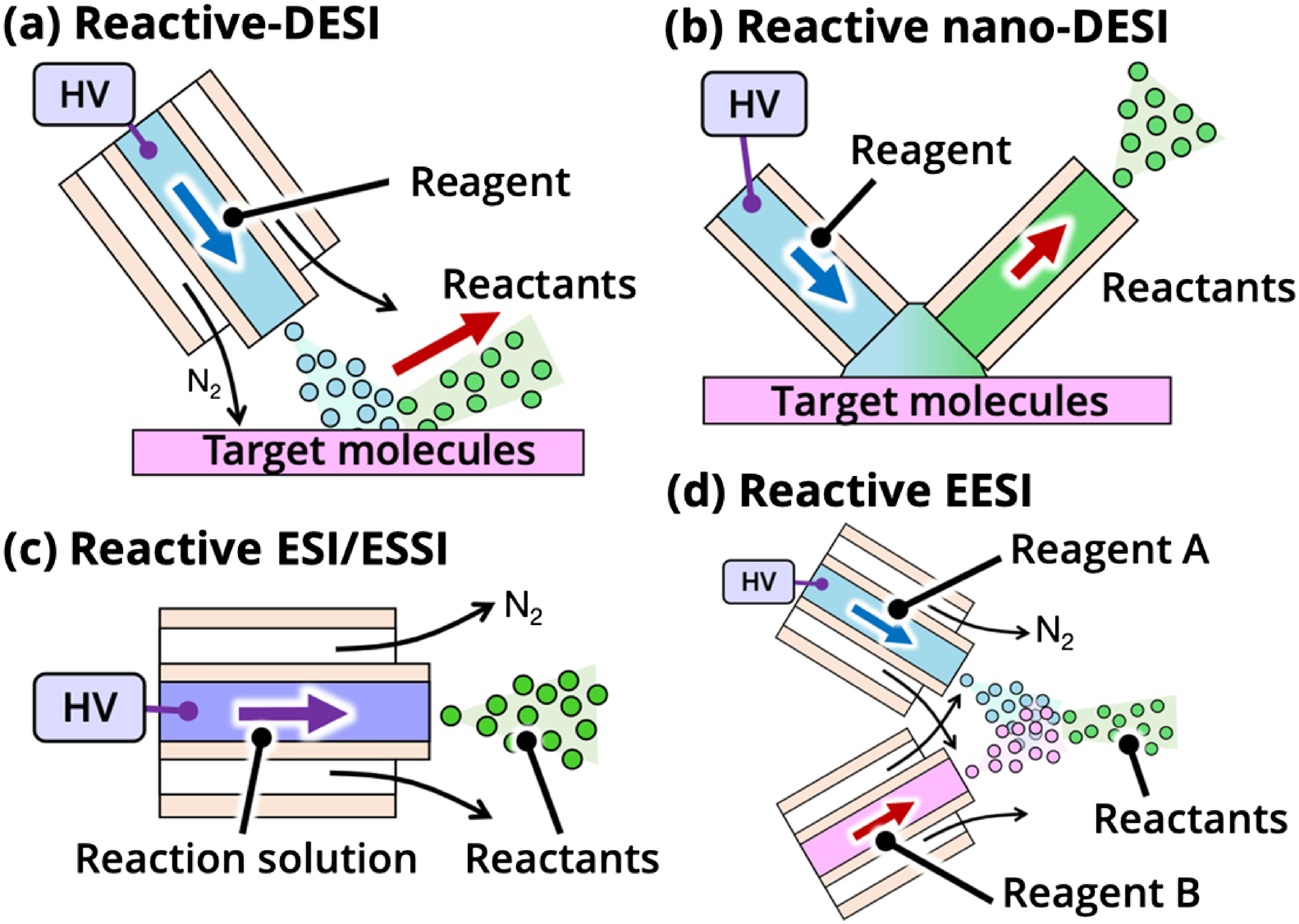
Fig. 13. Schematic of atmospheric pressure sampling ionization method utilizing derivatization reaction with charged droplets (a) Reactive DESI, (b) Reactive nano-DESI, (c) Reactive ESI/ESSI, (d) Reactive EESI.

The 2000s saw an increase in research on DLEI, and studies of derivatization reactions in charged droplets have been reported. DESI has been instrumental in the study of chemical reactions using charged droplets. The method of colliding charged droplets containing reagents with molecules on a substrate and rapidly measuring the resulting chemical reaction is known as reactive DESI. This approach has been used to study fast chemical reactions and reaction intermediates^183,184)^ (Fig. 13 (a)).

Girard’s derivatization using reactive DESI was reported 74 years after Girard’s reagent was first described. When a charged droplet of Girard’s T reagent solution was sprayed onto a thin steroid film on a PTFE substrate, the derivatization reaction occurred in seconds.^185)^ Although this method has not been applied to the imaging of biological tissues, this application will likely be developed in the future, given reports of highly sensitive MALDI imaging of steroids in biological tissues after derivatization with Girard’s T reagent.^186,187)^

MSI of biological tissues to date using reactive DESI includes the measurement of cholesterol in atheroma using betaine aldehyde,^188,189)^ lipids using dicationic compounds,^190)^ and malondialdehyde in rat spinal cord using dinitrophenylhydrazine.^191)^

In nano-DESI, derivatization of organic components on the substrate by adding Girard’s T reagent in a solvent (reactive nano-DESI) has been reported^192)^ (Fig. 13 (b)).

The combination of derivatization and DLEI thus holds promise for the highly sensitive measurement of molecules with low ionization efficiency.

### 6.2 Accelerating chemical reactions in small droplets

The integration of DLEI and selective derivatization requires an understanding of the chemical reactions that occur in microdroplets. This section describes studies to date on chemical reactions using MS.

The first attempts to study the mechanisms of organic and inorganic chemical reactions using ESI were reported in the 1990s.^193)^ Combining ESI with off-line or on-line chemical reactions has provided important insights into synthetic chemistry, including structural analysis of reaction products and intermediates, and deepened understanding of reaction pathways.

Atmospheric pressure ionization techniques for liquid samples were developed concurrently with research into DLEI in the 2000s. Mixing droplets of known molecular solutions in air by one of two approaches accelerates chemical reactions. In one method, a solution containing all the molecules of the reacting system is dropletized (Fig. 13(c)), and in the other method each molecular solution that makes up the reacting system is dropletized separately, then mixed by collision in air (Fig. 13(d)).

The first method uses ESI and electrosonic electrospray ionization (ESSI)^194)^ and is called reactive-ESI/ESSI. The ion source in ESSI, like DESI, is a capillary with a co-axial structure that generates charged droplets by flowing a solution through the inner channel and high-pressure nitrogen gas through the outer channel. Chemical reactions occur as the mixed solution dries, and ions are produced.

The second method is called reactive-EESI and uses extractive electrospray ionization (EESI).^195)^ Two ESSI ion sources are used: one for the sample solution, and the other for the solvent to generate the charged droplets. By colliding the two sprays, chemical reactions proceed in the droplets during the mixing and subsequent drying process, and the produced ions are measured.

Studies of the change in reaction products against time (μs to ms) between the formation of the charged droplet and its introduction into the mass spectrometer showed that charged droplets help accelerate chemical reactions, including the formation of hydrazine,^196)^ the demetallation of chlorophyll,^197)^ Hantzsch synthesis,^198)^ catalyst-free *N*-alkylation of indole,^199)^ synthesis of ribonucleosides,^200,201)^ acid-induced unfolding of cytochrome *c*, hydrogen-deuterium exchange in bradykinin^202)^ and angiotensin I,^203)^ and the Baeyer–Villiger reaction.^204)^ For more detailed descriptions, we refer the reader to comprehensive reviews.^205–207)^

**Figure 14:**
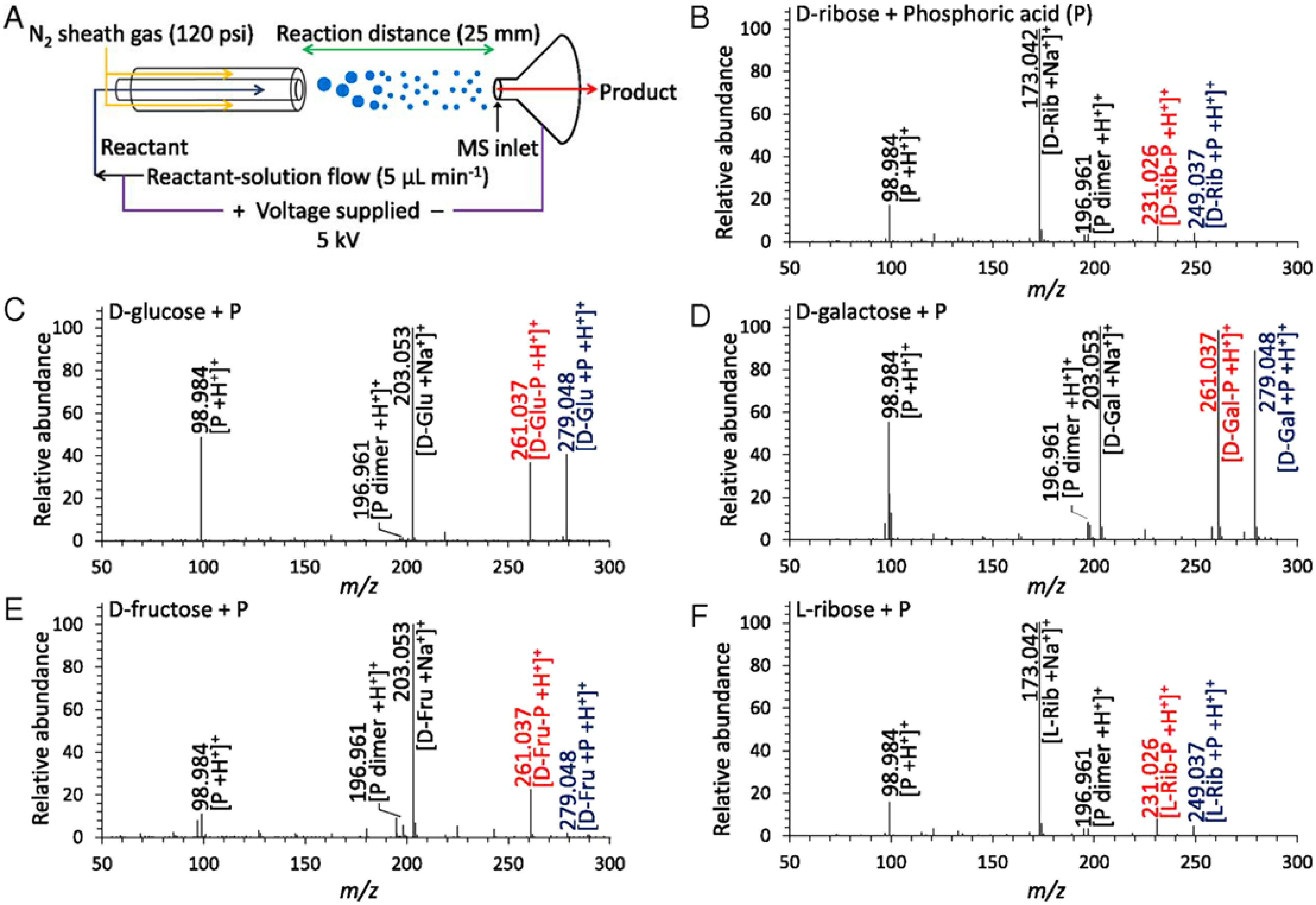
Fig. 14. Chemical reaction of sugar and phosphoric acid using reactive ESI. Reproduced with permission from Ref. 201. Copyright (2017) National Academy of Sciences.

As an example, Fig. 14 shows the results of the phosphorylation of sugar using reactive ESI. Chemical reactions that do not proceed in bulk solution of the mixture at room temperature spontaneously occur during drying of the charged droplets of the mixed solution, likely due to the negative change in Gibbs free energy.^201)^

Factors that specifically promote chemical reactions in charged droplets include the air–liquid interface,^197)^ the orientation and diffusion of molecules at the surface of the droplet, the change in concentration of reactive molecules during the drying process,^198,208)^ and changes in pH.^185,209)^

Multiple processes occur simultaneously in charged droplets, and thus new measurement methods and computational (physical) simulation^210)^ are important for understanding these processes. A method combining aerosol generation and ionization by DART (direct analysis in real time) was proposed to separate the reactions occurring in the droplets from those in the gas phase ions.^211)^ This method can be used to measure the gas–liquid interface effects of droplets and concentration effects in droplets. Another study reported the measurement of the concentration of a solute in picoliter droplets by Raman spectroscopy combined with an optical tweezer and whispering gallery mode resonance for aerosols.^212)^ The application of spectroscopic techniques to measurements of charged droplets will aid in understanding the dynamic changes in pH of charged droplets.

Future studies of charged droplets that mimic the intracellular chemical environment are expected to illuminate a connection between accelerated chemical reactions in droplets and the measurement of biomolecules by DLEI.

Studies to date have reported the mechanism of ionization and ionization suppression in charged droplets containing a limited number of molecules. Understanding how to apply these mechanisms to charged droplets containing a wide variety of molecules,^52)^ and how to preferentially derivatize specific components in these multi-component mixtures to achieve highly sensitive detection, will help advance our fundamental understanding of DLEI and its potential applications.

## 7. SUMMARY AND FUTURE PROSPECTS

This review described DLEI, a combination of solvent-based direct extraction and ESI, and related studies. The research and development of DLEI began in the early 2000s and is now in the expansion phase. The technology to precisely examine health conditions by capturing biomarkers localized in diseased tissues is beginning to find clinical applications that will continue to develop.

On the other hand, chemical information on living organisms obtained by DLEI is limited by the ion suppression effect, making comprehensive or quantitative measurements challenging. Minimizing the volume of liquid subjected to extraction and ionization is one approach to reduce ion suppression. Measuring a wide variety of chemical components will require further basic research on solvent selectivity, ionization processes of multiple components, and specific chemical reactions of charged droplets.

As the understanding of liquid–liquid phase separation in a picoliter cell space is considered to lead to a deeper understanding of biological activities, the investigation of spontaneous changes of chemical states in microdroplets will advance the measurement of multidimensional chemical information by using mass spectrometry and contribute to a deeper fundamental understanding of biosystems and biomedical applications.

## Notes

Mass Spectrom (Tokyo) 2021; 10(1): A0095

## Notes

This article is a secondary publication of *J. Mass Spectrom. Soc. Jpn*. 68: 59–74 (Japanese).
